# Assessment of the Variability in the Occurrence of PFAS in Fish Tissues from Selected Fisheries in the Baltic Sea

**DOI:** 10.3390/molecules29246029

**Published:** 2024-12-20

**Authors:** Joanna Kuc, Iwona Grochowalska, Maciej Thomas, Tamara Zalewska, Marta Rybka-Murat

**Affiliations:** 1 Faculty of Chemical Engineering and Technology, Cracow University of Technology, Warszawska 24, 31-155 Cracow, Poland; 2 Faculty of Natural Sciences, Jan Kochanowski University in Kielce, Stefana Żeromskiego 5, 25-369 Kielce, Poland; iwona.grochowalska@ujk.edu.pl; 3 Faculty of Environmental Engineering and Energy, Cracow University of Technology, Warszawska 24, 31-155 Cracow, Poland; maciej.thomas@pk.edu.pl; 4 Institute of Meteorology and Water Management, National Research Institute, Waszyngtona 42, 81-342 Gdynia, Poland; tamara.zalewska@imgw.pl (T.Z.); marta.rybka-murat@imgw.pl (M.R.-M.)

**Keywords:** PFAS, LC–ESI–MS/MS, persistent organic pollutants, xenobiotics, bioaccumulation, Baltic Sea monitoring

## Abstract

In this study, the results of a comprehensive assessment of the variability in the occurrence of ten perfluorinated compounds (PFAS) in fish tissues originating from 2014 to 2019 from six fisheries in the Baltic Sea are presented. A total of 360 fish samples of three species (perch, herring and flatfish) were analysed. For the determination of PFAS, both linear and branched stereoisomers, LC–ESI–MS/MS technique preceded by simultaneous SPE isolation was validated and applied. The total concentration of all determined PFAS compounds shows that the highest levels were observed in the Szczecin Lagoon (4.8 ± 0.7 µg/kg) and the lowest in the Pomeranian Bay (1.9 ± 0.1 µg/kg). In most samples, the dominant compound was perfluorooctane sulfonic acid (PFOS). The present research enabled the assessment of the variability in the occurrence of PFAS stereoisomers in marine fish.

## 1. Introduction

A balanced and varied diet containing not only nutrients but also substances that have a beneficial effect on human health is essential to prevent degeneration, ageing and other processes unfavourable to health, including those resulting from environmental pollution. In this context, the consumption of fish is recommended for their beneficial cardioprotective properties because of the optimal ratio (1:1.5:1) of unsaturated fatty acids to monounsaturated fatty acids (MUFAs) and polyunsaturated fatty acids (PUFAs) [1] the high content of proteins and mineral substances [2]. However, fish tissues may be prone to the accumulation of different xenobiotics, which occur in aquatic ecosystems. Therefore, fish are excellent bioindicators of contamination, which makes it possible to conduct a risk assessment for humans [3]. The accumulation and biomagnification of organic pollutants in fish tissues affect the health of consumers [4]. The level of xenobiotic pollution is an important parameter for assessing the quality of the natural environment and the ecological status of fisheries.

Over 4700 per- and polyfluoroalkyl compounds (PFAS) containing a −CnF2n– (n ≥ 3) or −CnF2nOCmF2m– (n and m ≥ 1) moiety have been synthesised and classified as endocrine disruptors according to the Organization for Economic Cooperation and Development (OECD) [5]. The names perfluorinated organic compounds (PFOC), perfluorinated chemicals (PFCs) and, more often, perfluorinated alkylated substances or per- and polyfluoroalkyl substances (PFAS) are a large class of xenobiotic compounds in which hydrogen atoms bound to carbon in the alkyl chain have been replaced by fluorine, which results in the strongest polar covalent carbon–fluorine bond in organic chemistry [6,7]. The EPA now uses “per- and polyfluoroalkyl substances” (PFAS) rather than “perfluorinated chemicals” (PFCs) to describe this group of chemicals [8]. PFAS are thermally and chemically stable and extremely persistent in the environment. In turn, the presence of polar functional groups results in hydrophilic and lipophilic properties, which makes PFAS very mobile and stable in the environment [4]. Because of these properties, these compounds are called “forever chemicals”. PFAS have been used since the 1940s in the military [9] industry and for protection, especially in fire-fighting materials [10,11], aviation hydraulic fluids [12], in industry for the production of paints and sprays [13], insulating and impregnating for building materials [14], and insecticide formulations [15], as well as in everyday consumer products such as clothing fabrics, leather, carpets [16] and even cosmetics [17]. This versatile use contributes to the inevitable exposure of animals and humans to the harmful effects of PFAS compounds, which are considered immunological and endocrine disrupters and have health implications associated with liver toxicity [18,19,20,21,22]. The harmful effects of PFAS on the human body were known in 1970 by companies producing PFAS; however, they were carefully cut down for at least 21 years until they were made public as a result of court cases [23].

Within the last decade, PFAS has been increasingly mentioned among environmental contaminants due to their presence in various types of abiotic and biotic matrices and in all types of environmental samples. Numerous studies have confirmed their presence in surface water, groundwater, soil, sediments, air, plants and even animal tissues [4,24,25,26,27,28,29,30,31].

The Stockholm Convention regulates the global elimination of perfluorooctane sulfonic acid and its derivatives (PFOS) and perfluorooctanoic acid (PFOA), its salts and PFOA-related compounds. Under the EU’s Persistent Organic Pollutants (POPs) Regulation, PFOS has been restricted since 2009, and PFOA has been banned since 2020. Moreover, beginning in 2020, the European Food Safety Authority (EFSA) concluded that PFOS, PFOA, PFNA (perfluorononanoic acid) and PFHxS (perfluorohexanesulfonic acid) may have adverse effects on the immune system, endocrine function and carcinogenicity. Currently, the maximum level of these four PFAS contaminants in food is regulated by edible meat, fishery products and eggs, which are considered individual compounds and their sum [32]. Furthermore, the EU Commission recommended monitoring the concentrations of selected PFAS in fruit, vegetables, mushrooms, starchy roots and tubers, and milk [33].

Over the last two decades, many publications and specifications from analytical equipment manufacturers have been published on the determination of PFAS compounds via the LC–MS/MS method [34,35,36,37]. However, these compounds may occur in the natural environment in two forms, linear and branched, and their analysis has been mentioned as problematic [38,39,40]. Animal studies have shown that the linear forms of PFOS and PFOA have stronger binding affinities to serum albumin than the branched forms [41]. Scientists hypothesise that the branched isomers of these compounds may degrade differently in the environment and have different toxic effects on animals and humans than the linear forms [29]. Different levels of exposure to these forms of PFAS may lead to different health effects for humans and animals, e.g., body weight reduction in rats and mice relative to digestible food [42].

Therefore, it is very important to determine a method that allows simultaneous analysis of the linear and branched forms of PFAS compounds. There is still little research in this area, and the results of the research carried out over several years by the authors of this publication, using a method enabling the distinction between linear and branched forms, supplement the existing knowledge.

## 2. Results and Discussion

### 2.1. LC–MS/MS Analysis

**Figure 1:**
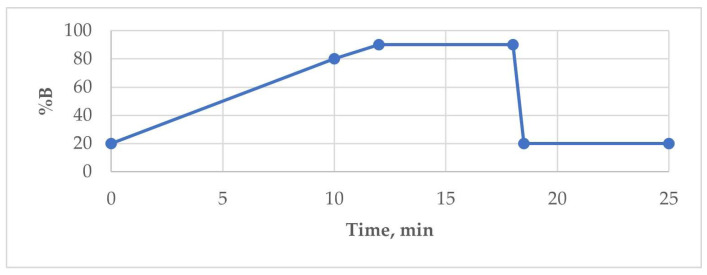
Gradient plot of the mobile phase.

Following the infusion of analytes into the mass spectrometer, the mass spectrum of PFAS showed an ion [M − H]^−^, with major fragments. The *m*/*z* values of the precursor and product ions are shown in Table 1. The interface parameters were as follows: nebulising gas, 2 L/min; drying gas, 10 L/min; DL temperature, 260 °C; and capillary voltage, 2.0 kV. A gradient flow (Figure 1) of the mobile phase was used, with the following composition: 10 mM ammonium acetate in water (A) and 10 mM ammonium acetate in methanol (B). The column temperature was 40 °C, and 10 μL of sample was injected.

The EU Regulation [32] does not require separate determination of PFAS stereoisomers; it is stated that the maximum level applies to the sum of linear and branched forms, regardless of whether the analytical method allows them to be separated. Despite this, researchers suggest implementing methods for determining individual PFAS stereoisomers because their different physicochemical properties influence their degradation, toxicokinetic and bioaccumulation abilities [40].

**Figure 2:**
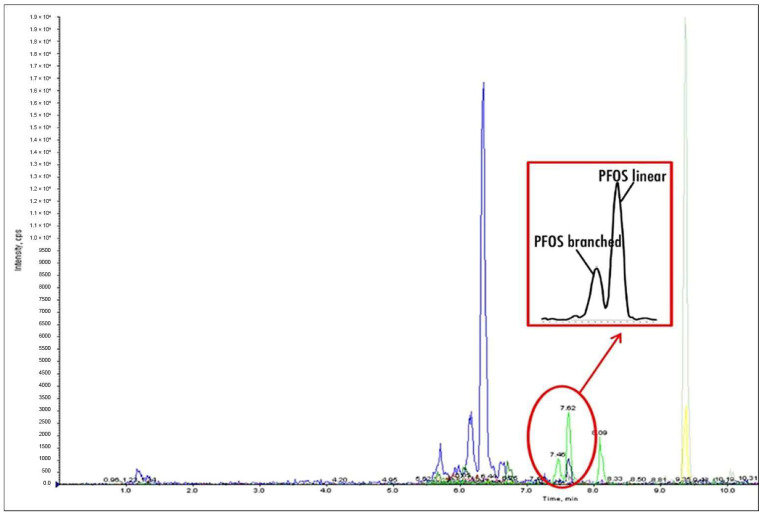
Linear and branched forms of PFOS.

The analytical method developed by the authors in 2014 allowed for the simultaneous determination of the linear and branched forms of selected PFAS compounds. Figure 2 shows an example chromatogram that illustrates the occurrence and separation of these two stereoisomers for PFOS. The chromatogram was obtained during the analysis of one of the perch samples from Szczecin Lagoon. The linear form of PFOS gives a signal more than twice as high as that of the branched form, which confirms the results of other researchers [41].

### 2.2. Validation Data

The quantitation of PFAS was evaluated via five-point calibration curves, which ranged from 0.01 µg/kg to 2.0 µg/kg. The R2 value of the calibration curve for all the compounds was greater than 0.998, depending on the target analyte.

The described research has been carried out since 2014, and the recommendations and regulations to which we compare the EU results have appeared only since 2022. Nevertheless, the PFAS determination method developed in 2014 is so precise and accurate and intended for analyses at trace concentration levels that we meet the limits recommended by the European Commission [33] (Commission Recommendation (EU), 2022). Our study revealed the LLOQ for perfluoroalkyl carboxylic acids (PFCAs) at 0.03 µg/kg and for perfluoroalkane sulfonates (PFSAs) at 0.1 μg/kg. The aforementioned EU Commission Recommendation gives the value of the LLOQ as 0.1 μg/kg for PFOS, PFOA, PFNA and PFHxS in fish meat. In our tests, the LLOQ for PFOA and PFNA was much lower, whereas for PFOS and PFHxS, it was equal to the recommended LLOQ.

The selectivity of the method was assessed via systematic analysis of blank samples. No interferences were observed during the retention time of each target analyte. Quality controls (QCs) were used to assess the precision, accuracy and stability of the samples. The QCs were prepared in the same matrix as the study samples to be assayed with the validated method. Low QCs concentration was 0.03 µg/kg for perfluoroalkyl carboxylic acids (PFCAs) and 0.1 μg/kg for perfluoroalkane sulfonates (PFSAs). High-QCs concentration was 2.0 µg/kg for all PFAS. The coefficient of variation (CV) was <19% and the recovery ranged from 72 to 108%. According to the Data Review and Validation Guidelines for Perfluoroalkyl Substances (PFASs), the CV and recovery values for target analytes are <20% and 70–130%, respectively [43]. In turn, in the Data Validation Guidelines, the CV value should be <20%, and the recovery should be <200% [44]. These values concern the analysis of water samples, but the values obtained in this study for fish samples also fall within the acceptable level of the validation criteria.

Carry-over in the blank samples was <5% of the LLOQ of native standards, and the CV determined for QC samples to evaluate matrix effects was also <5%, which gives acceptable results in relation to the guidelines for the analysis of compounds in bioanalytical methods [45].

### 2.3. Concentration of PFAS in the Fish Samples

The contents of individual PFAS in the fish samples from a given fishery from 2014 to 2019 are given in Table 2. Table 3, Table 4 and Table 5 list the minimum (Min) and maximum (Max) values and the range (R) as the difference between the largest and the smallest values of a statistical feature in the set. To illustrate the relationships between individual fisheries in subsequent years, the samples were grouped, and the mean concentrations of the determined compounds, as well as the total PFAS concentrations, were considered. Additionally, the data were analysed according to the species of fish examined.

PFBA, PFNA, PFDA, and PFOS were determined for each fish sample analysed. PFHpA and PFBS were identified the least frequently. In approximately 40% of all the samples, the concentration of these compounds was <LOQ, and in some cases, in a given year and fishery, these analytes were detected in only one sample. This is marked in Table 2 as “µ” and means the uncertainty of a single measurement.

The analysis of the results in Table 3, Table 4 and Table 5 revealed that the largest ranges between the highest and lowest concentration values were obtained for PFOS, PFBA and PFNA. The smallest dispersion of results occurs for PFBS, PFHxS and PFHpA. In this case, the place of fishing or the year of sampling did not matter. Therefore, the larger range between the largest and smallest concentrations may result from the different bioaccumulation, biomagnification and biodilution of individual PFAS substances. The metabolic differences in accumulation and trophic magnification depend largely on the living environment of a given fish species as well as permanent or periphery sources of exposure to pollution [46,47,48]. Trophic magnification factors (TMFs) may also be influenced by the structure of food webs when there are large numbers of fish, which may weaken pollutant flows [49]. Moreover, variances in protein content in the tissues of fish species can result in variability in bioaccumulation [4].

#### 2.3.1. Comparison of Total PFAS Content in the Fishery

**Figure 3:**
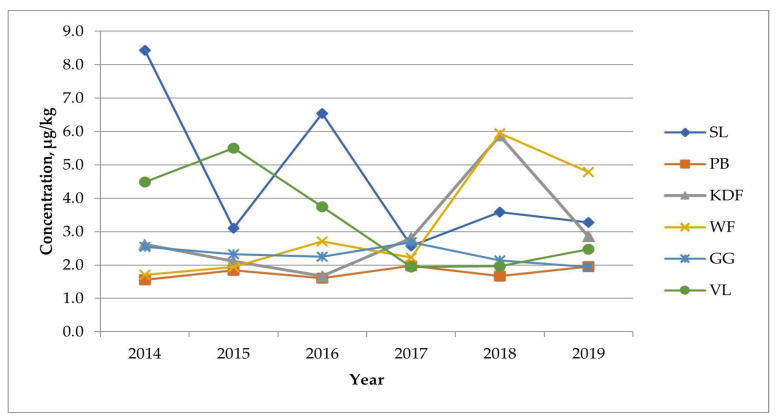
Variability in total PFAS concentrations in a given fishery over time.

**Figure 4:**
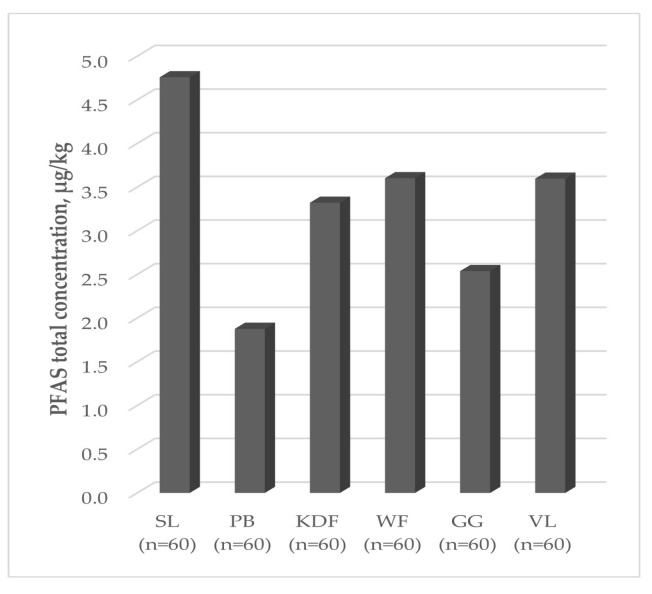
Total PFAS concentrations in a given fishery.

To assess the variability of total PFAS concentration levels in particular years in a given fishery, Figure 3 was prepared. The total concentrations of PFAS in all the tested samples over six years are shown in Figure 4.

The concentration of total PFAS over six years did not change significantly in the Gulf of Gdańsk (GG) or Pomeranian Bay (PB), where the samples tested were perch fish. In the case of herring fish caught in the Władysławowski (WF) and Kołobrzesko-Darłowski (KDF) fisheries, a significant increase in the concentration of total PFAS has been observed since 2017, whereas in the Vistula Lagoon (VL) after 2015, the PFAS level decreased significantly. In the Gulf of Gdańsk (GG), where flatfish were collected, concentration changes were initially abrupt, and from 2017, a decrease was observed, rather than slight variability in subsequent years. The fact that the same relationships are characteristic of the same species of caught fish seems to be highly interesting. It would seem that the decreases, increases or lack thereof in the total PFAS correlate with the fish species, but such a clear statement would be a far-reaching simplification despite the large population of samples tested. Only several years of research on other fish species and comparisons of correlations with the source of pollution at the fishing site would provide a broader view of this hypothesis.

The total concentrations of all the determined PFAS compounds over six years (Figure 4) revealed that the highest levels were observed in Szczecin Lagoon (4.8 ± 0.7 µg/kg), and the lowest was observed in Pomeranian Bay (1.9 ± 0.1 µg/kg). This can be explained by the fact that the main source of pollutants introduced into the Szczecin Lagoon is Odra runoff, and the Lagoon serves as a buffer reservoir protecting the waters of Pomeranian Bay against the impact of pollutants brought from the Odra catchment area.

#### 2.3.2. Comparison of Individual PFAS Contents by Species

**Figure 5:**
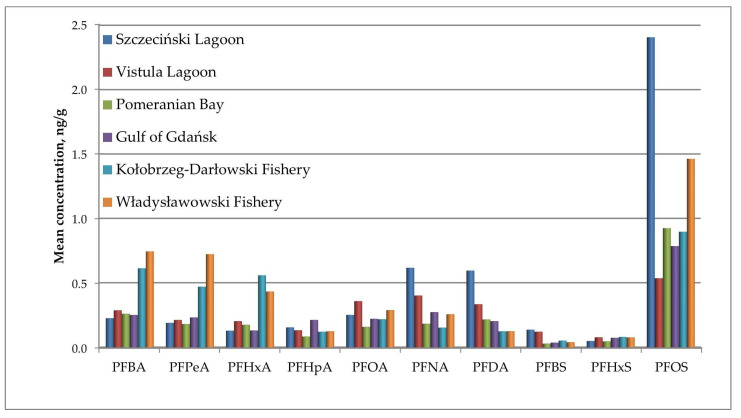
The concentration of individual PFAS in all samples from a given fishery.

**Figure 6:**
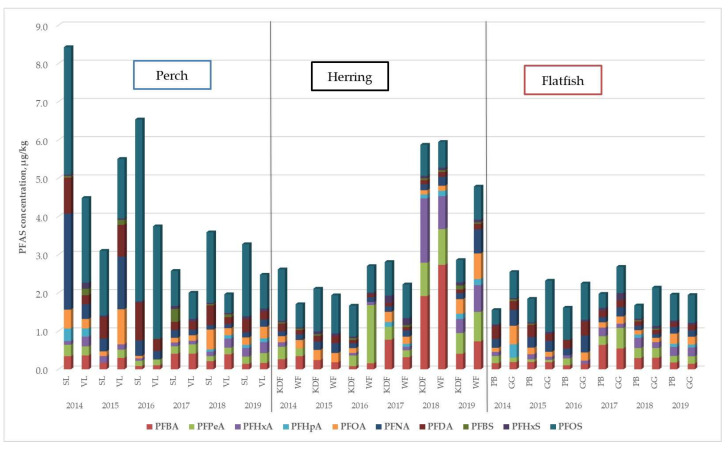
Concentration of PFAS in fish species.

A comparison was made of the degree of contamination of fisheries with individual PFAS compounds. The contents of individual compounds in all the fish samples from a given fishery are shown in Figure 5. A comparison of the results by fish species is shown in Figure 6.

In most samples, the dominant compound was PFOS, and the highest concentration was recorded in Szczecin Lagoon (2.4 µg/kg), which correlates with the discussion in point 3.3.1. The Agency for Toxic Substances and Disease Registry reported that the half-lives of PFAS vary for individual compounds and depend on the animal species. For example, the estimated half-life of PFOS in humans is up to 27 years, but in experimental animals, it is much shorter; however, how much shorter it is not stated [50]. Moreover, one PFAS compound, perfluorooctane sulfonamide (FOSA), can transform into perfluorooctanesulfonate acid (PFOS), perfluorooctanoic acid (PFOA), and shorter-chain PFCAs [51], which results in increased exposure to PFOS. FOSA derivatives are composed of perfluorinated carbon chains and nonfluorinated functional groups and can be transformed into more toxic PFCAs or perfluoroalkyl sulfonic acids (PFSAs) via biological or abiotic processes [52,53].

Perch, herring and flatfish are key fish species in the Baltic Sea and lagoon ecosystem. All species are used in assessing the state of the marine environment due to their prevalence [54,55]. In this study, the lowest PFAS concentrations were detected in flatfish, the highest were detected in perch from 2014 to 2016, and the lowest PFAS concentrations were detected in herring from 2018 to 2019. Flatfish had comparable results in all years, with PFOS being the most abundant, except for 2017, when PFBA was more abundant than PFOS in fish collected from Pomeranian Bay. In the case of perch fish, the highest concentration was recorded in 2014, which consisted mainly of PFOS and PFPeA. In 2016, high total concentrations of PFAS were subsequently observed in Szczecin Lagoon, with PFOS dominating.

In samples of herring from the Władysławowski and Kołobrzesko-Darłowski fisheries in the years 2014–2017, comparable results were obtained and similar to those in the case of flounder. In 2018, an increase in the total PFAS value was recorded in herring samples, and unlike in other years, PFBA, PFHxA and PFPeA were the main compounds that caused an increase in the total PFAS level. Perhaps it is related to illegal discharges of pollutants into the Baltic Sea, as HELKOM [56] reported in 2018, a total of 155 observations of spills into the Baltic Sea were reported, 40% of which concerned mineral oils (non-PFAS) and the remaining 60% identified as other substances (unidentified) that may pose a threat to the marine environment.

#### 2.3.3. Comparison of the Concentrations of the Four Regulated Compounds Determined in These Studies

**Figure 7:**
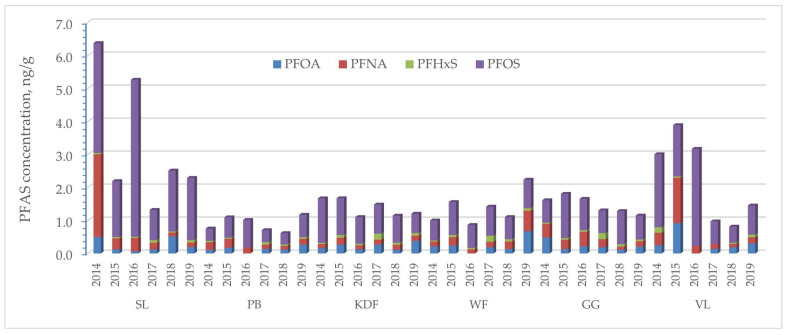
Concentration of PFOS, PFOA, PFNA and PFHxS in the fish samples.

In the EU Regulation [32], the maximum level applied to the fish samples depends on the species and consumer group. The limit values applicable to infants and young children are much more restrictive. Generally, the limitations are 2–35 μg/kg for PFOS, 0.2–8 μg/kg for PFOA, 0.5–8 μg/kg for PFNA, 0.2–1.5 μg/kg for PFHxS and the sum of those four in the range of 2–45 μg/kg. The EU Commission also issued recommendations regarding the testing of another 18 compounds from the PFAS group that could be as toxic as PFOS, PFOA, PFNA and PFHxS, but they differ in chemical structure [33]. The average concentrations of the four compounds in individual fisheries over six years are shown in Figure 7.

The highest values of the sum of PFOS, PFOA, PFNA and PFHxS were recorded for perch from the fisheries of Szczecin Lagoon in 2014 and 2016 and from Vistula Lagoon in 2014, 2015 and 2016. PFOS was dominant in all samples, regardless of the year of sampling, despite restrictions in the EU since 2009 [57], which resulted from the persistent nature of this compound and its ability to bioaccumulate in the natural environment, including fish.

Among those recommended for testing are also those determined in these studies (PFBA, PFPeA, PFHxA, PFHpA, PFDA, PFBS). These subsequently introduced compounds result from scientific reports regarding the harmful effects of individual compounds from the group of eternal chemicals. For example, PFHpA and its salts are known to have probable serious effects on human health and the environment and have been listed as substances of high concern by the European Union because of their toxic properties since January 2023 [58] (ECHA, 2023).

**Figure 8:**
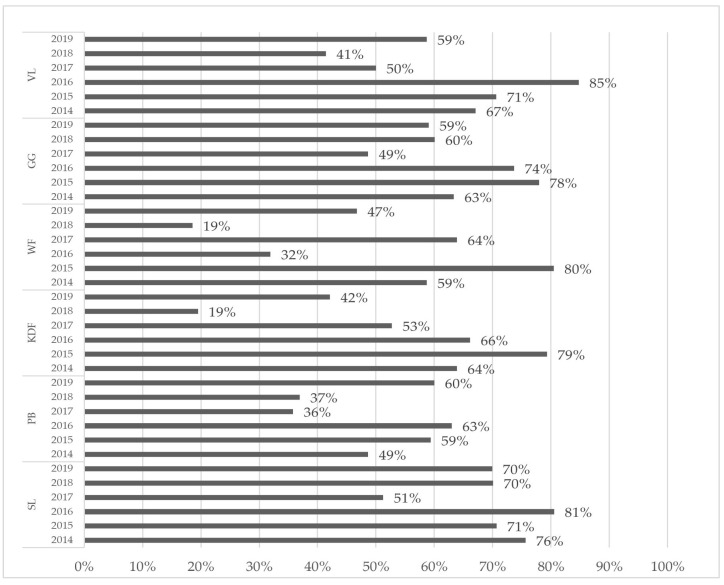
The percentage of the sum of four regulated compounds in relation to the sum of PFAS was determined in these studies.

To demonstrate the differences between the total concentrations of the four compounds required for testing under EU law and the concentrations obtained in this study, the percentages are presented in Figure 8.

Figure 8 shows that the analysis of only four compounds led to an underestimation of the risk posed by other compounds from the PFAS group. This may be a reason to consider implementing the EU recommendation [33] or considering whether it is worth monitoring more PFAS compounds in food, such as those for drinking water listed in the Water Directive [59]. Moreover, the toxic effects of PFAS on marine and freshwater algae may suggest the existence of various toxicity mechanisms for individual compounds [60].

## 3. Materials and Methods

### 3.1. Sample Collection and Preparation

**Figure 9:**
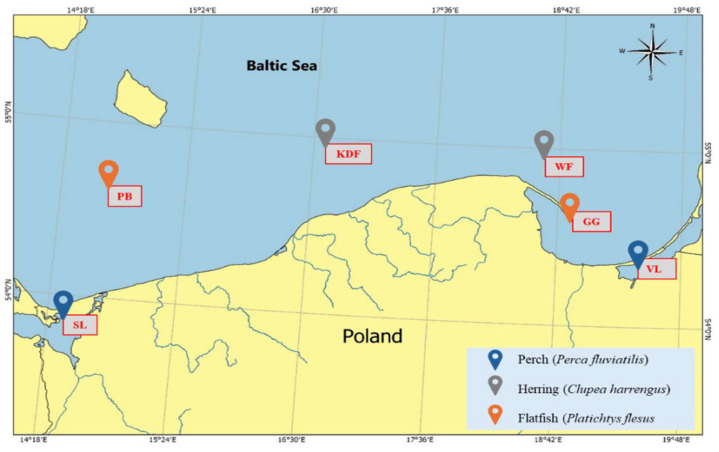
Fishery locations.

The fish samples were obtained from commercial fishing carried out each year from September to November each year from 2014 to 2019 at 6 locations (Figure 9). Herring (*Clupea harrengus*) samples were obtained from the areas of the Kołobrzeg-Darłowski (KDF) and Władysławowski (WF) fisheries; flatfish (*Platichtys flesus*) samples were obtained from the Gulf of Gdańsk (GG) and Pomeranian Bay (PB), whereas perch (*Perca fluviatilis*) samples were obtained from Szczeciński Lagoon (SL) and Vistula Lagoon (VL).

In the first step of the preparation of the fish samples, ichthyological analysis was included. The length, weight, age, sex and development stage of the gonads were determined. For sex and gonad development stage determination, macroscopic observations of gonads were carried out. The 8-point Meier gonad maturity scale was used. Only females from which muscle samples were taken for the study. The fish were transported under refrigerated conditions to the laboratory, and meat tissue was removed from each sample. The samples were lyophilised before analyte extraction and quantitative analysis of harmful compounds specified in the Baltic Sea monitoring system [61]. The monitoring system was coordinated by the Chief Inspectorate of Environmental Protection and financed by the National Fund for Environmental Protection and Water Management. The abovementioned monitoring studies only require the determination of the concentration of one substance, perfluorooctane sulfonic acid (PFOS). Nevertheless, the authors of this publication conducted additional research to a broader extent, and in addition to PFOS, they determined an additional 9 compounds from the PFAS group in the fish samples obtained from 2014 to 2019.

### 3.2. Chemicals

Water, acetonitrile and methanol hypergrade for LC–MS (LiChrosolv^®^) and ammonium acetate for LC–MS (LiChropur™) were purchased from Merc KGaA, Darmstadt, Germany. Standard solvents were prepared in polypropylene vials manufactured from high-quality polypropylene and known as PTFE-free (Thermo Scientific™, Thermo Fisher Scientific Inc., Waltham, MA USA).

The levels of selected perfluoroalkyl carboxylic acids (PFCAs), such as perfluorobutanoic acid (PFBA), perfluoropentanoic acid (PFPeA), perfluorohexanoic acid (PFHxA), perfluoroheptanoic acid (PFHpA), perfluorooctanoic acid (PFOA), perfluorononanoic acid (PFNA) and perfluorodecanoic acid (PFDA), as well as perfluoroalkane sulfonates (PFSAs), such as perfluorobutane sulfonate (PFBS), perfluorohexane sulfonate (PFHxS) and perfluorooctane sulfonate (PFOS), were determined via an LC–ESI–MS/MS method. As internal standards, mass-labelled solution mixtures were used. Certified analytical standards of all PFAS, both native and mass-labelled, were obtained from Wellington Laboratories, Inc., Guelph, ON, Canada. Table 6 summarises the analytical standards used in these studies.

### 3.3. Extraction Procedure

The extraction procedure was introduced on the basis of the enrichment of the analyte with simultaneous isolation from the matrix via solid phase extraction (SPE). To achieve this goal, 100 mg of lyophilised muscle from each fish sample was extracted via ultrasound-assisted extraction (30 min) with acetonitrile. The sample was centrifuged at 4000 rpm for 5 min (MPW Med. Instruments, Warsaw, Poland), and the supernatant was transferred to a new clean vial. The mixture was then evaporated in a stream of nitrogen (99,999%, Air Products, Cracow, Poland) to a volume of 1 mL. In the next step, Oasis WAX, 3 mL of 1% NH3_(aq)_ in a methanol cartridge (Waters™, Milford, MA, USA) was used. The Oasis WAX SPE column is a mixed-mode of weak anion exchange and reversed-phase polymer that delivers purity and quality and enables low-level quantification of PFAS [63]. After evaporation in a nitrogen stream to a volume of 50 μL, the sample was diluted with methanol to 100 μL and then filtered through a 0.2 μm syringe filter with a nylon membrane (Sigma Aldrich, Burlington, MA, USA). The internal standards were used to compensate for matrix effects and instrumental bias.

### 3.4. Chromatographic Separation and Detection

In this study, the LC–ESI–MS/MS method was used for qualitative and quantitative analysis of selected PFAS, both in linear and branched forms. Simultaneous quantification requires ensuring high analytical method efficiency and sensitivity. Before proceeding to quantitative analysis in selective multiple reaction monitoring (MRM) mode, the electrospray ionisation (ESI) source was optimised. In the first stage, the parameters for precursor ions were optimised using the monoisotopic masses of PFAS compounds as references. Monoisotopic mass is most often used for molecules containing larger numbers of atoms with excess mass. In those cases, the difference between the monoisotopic mass and the average mass increases as the compound mass or relatively abundant heavy isotope increases. If the isotopes for large compounds are not resolved, the peak centroid corresponds to the average molecular mass [64]. For example, for some fluorinated compounds (fluorinated phosphagens), the monoisotopic peak is more intense at far higher masses than for comparable hydrocarbon chains [65]. In the next stage, fragmentation was carried out to isolate product ions and the maximum collision energy that occurred for each MRM pair was chosen.

A high-performance liquid chromatography from Shimadzu UFLC30 Nexera (Kyoto, Japan) was connected to a QTRAP 3200 mass spectrometer from Sciex (Framingham, MA, USA). Chromatographic separation was carried out on a Kinetex 2.6 μm XB C18 100A column (100 × 2.1 mm; Phenomenex Inc., Torrance, CA, USA) with a SecurityGuard Ultra C18 precolumn and delay Kromasil C18 column (Phenomenex Inc., Torrance, CA, USA). The delay column was installed between the sample injector mixer and the mobile phase mixer to isolate interferences related to possible PFAS compounds, e.g., in solvents and LC system elements. The use of this column ensured accurate measurement of PFAS in the sample.

### 3.5. Method Validation

The LC–MS/MS method was validated with respect to selectivity, sample stability, precision and accuracy, matrix effects, carry-over and recovery of analytes. All procedures conducted during method validation were performed using blank samples spiked with native and isotopic internal standards of PFAS. In the first step, solvent blanks (methanol, water and acetonitrile) were analysed to control background interferences from the mobile phase and instrument. For the purpose of method validation, blank samples were prepared and spiked with analytes via reference standard solutions. Solutions of calibration standards and low- and high-quality control samples (QCs) were also prepared.

To evaluate the selectivity of the analytical method, representative blank samples were analysed. To ensure high selectivity, the response of the blank must be less than the response of the lower limit of quantification (LLOQ).

The calibration curve for five different levels of PFAS was assessed by plotting the ratio of the analyte peak area to its concentration while accounting for the internal isotopically labelled standards. The evaluation was based on the coefficient of determination (R^2^).

Accuracy and precision were determined on the basis of analyses of the spiked sample in five replicates each. The accuracy of the method was expressed as the percentage of the difference in the deviation between the nominal concentration and the calculated concentration, and the precision was expressed as the coefficient of variation (CV). The recovery (%) of analytes was determined by comparing the results of real samples with those of enriched samples. To assess interference caused by the presence of other substances in the sample that may interfere with the analysis (matrix effect), 3 replicates of low and high QCs were analysed, and the CV was assessed.

When developing a method, carry-over (changes in the measured concentration resulting from the analysis of residue from a previous sample) must be assessed and minimised. In this research, carry-over was assessed during validation by analysing blank samples after analysing calibration standards at the upper limit of quantification (ULOQ).

## 4. Conclusions

PFAS are widespread in biotic and abiotic environments. They are commonly referred to as “forever chemicals” because of their use in many industries and consumer products and their properties typical of persistent organic pollutants. These harmful chemicals accumulate in living organisms and have the ability to biomagnify at relatively high trophic levels. Therefore, monitoring PFAS concentrations in environmental samples is necessary and required by the US and EU regulations regarding drinking water, surface water and food. The latest scientific reports on the toxicokinetic differences of PFAS stereoisomers make it necessary to develop and implement appropriate, sensitive methods for determining them via routine laboratory analysis. Risk assessment for ecological systems requires the selection of bioindicator species on the basis of ecotoxicological studies. This will enable us to obtain data on the effects of exposure to PFAS in environmental matrices and predict the potential for bioaccumulation and biomagnification, both for individual compounds and for the sum of PFAS.

The analysis of 360 fish samples from perch, herring and flatfish carried out between 2014 and 2019 allowed for an effective assessment of the variability in concentrations of individual PFAS compounds in correlation with specific sampling points. Research has shown that Szczecin Lagoon is most polluted by various chemicals, which is related to the Oder tributary and relatively shallow funnel-shaped formation, which is a cluster of pollution. The results revealed that in all the tested samples, PFBA, PFNA, PFDA and PFOS were above the quantification limit, with significant dominance of PFOS. Moreover, to create future legal regulations, food monitoring of a larger number of PFAS compounds (not just four) should be considered. It also seems reasonable to consider the separation and determination of both forms of PFAS stereoisomers—linear and branched.

## Figures and Tables

**Table 1:** Precursor and product ions of PFAS compounds.

Compound	Precursor Ion, *m*/*z*	Product Ion, *m*/*z*
PFBA	212.80	169.20
PFPeA	262.80	218.95
PFHxA	312.85	269.00
PFHpA	362.85	318.95
PFOA	412.85	368.95
PFNA	462.95	418.95
PFDA	512.85	468.90
PFBS	299.10	223.25
PFHxS	398.85	79.90
PFOS	498.95	80.00
MPFBA	217.20	172.30
MPFHxA	315.30	270.20
MPFOA	417.30	372.20
MPFNA	467.90	423.20
MPFDA	514.90	470.10

**Table 2:** Mean PFAS concentrations in the fish samples.

Fishery	Year (No of Samples)	Mean Content ± SD, µg/kg																													
		PFBA			PFPeA			PFHxA			PFHpA			PFOA			PFNA			PFDA			PFBS			PFHxS			PFOS		
**SL—Szczecin Lagoon**	2014 (n = 10)	0.3	±	0.2	0.3	±	0.1	0.10	±	0.02	0.33	±	0.02	0.5	±	0.2	2.5	±	1.8	0.9	±	0.5	0.05	±	0.01	0.04	±	0.01^µ^	3	±	1
	2015 (n = 10)	0.2	±	0.1	<LOQ			0.2	±	0.1	<LOQ			0.12	±	0.02	0.3	±	0.1	0.6	±	0.2	<LOQ			0.04	±	0.01	1.7	±	0.4
	2016 (n = 10)	0.09	±	0.02	0.14	±	0.04	0.06	±	0.01^µ^	<LOQ			0.06	±	0.01^µ^	0.4	±	0.1	1.0	±	0.2	<LOQ			0.03	±	0.01	5	±	1
	2017 (n = 10)	0.4	±	0.3	0.2	±	0.1	0.10	±	0.03	<LOQ			0.1	±	0.1	0.2	±	0.1	0.2	±	0.1	0.3	±	0.2	0.08	±	0.03	0.9	±	0.3
	2018 (n = 10)	0.2	±	0.1	0.13	±	0.04	0.12	±	0.05	0.06	±	0.01^µ^	0.5	±	0.2	0.1	±	0.1	0.5	±	0.2	0.03	±	0.01	0.03	±	0.01^µ^	1.8	±	0.6
	2019 (n = 10)	0.1	±	0.1	0.2	±	0.1	0.2	±	0.1	0.08	±	0.02	0.2	±	0.1	0.14	±	0.04	0.3	±	0.1	<LOQ			0.07	±	0.03	1.9	±	0.6
**PB—Pomeranian Bay**	2014 (n = 10)	0.16	±	0.04	0.2	±	0.1	0.11	±	0.05	<LOQ			0.11	±	0.04	0.2	±	0.1	0.3	±	0.1	<LOQ			0.04	±	0.01	0.4	±	0.1
	2015 (n = 10)	0.19	±	0.05	0.07	±	0.01^µ^	0.14	±	0.04	<LOQ			0.2	±	0.1	0.3	±	0.3	0.3	±	0.3	0.03	±	0.01^µ^	0.03	±	0.01	0.6	±	0.1
	2016 (n = 10)	0.10	±	0.03	0.2	±	0.1	0.08	±	0.01	<LOQ			<LOQ			0.2	±	0.1	0.2	±	0.1	<LOQ			<LOQ			0.8	±	0.2
	2017 (n = 10)	0.6	±	0.5	0.2	±	0.1	0.2	±	0.1	<LOQ			0.14	±	0.05	0.1	±	0.1	0.2	±	0.2	<LOQ			0.07	±	0.02	0.4	±	0.1
	2018 (n = 10)	0.3	±	0.1	0.3	±	0.1	0.26	±	0.03	0.09	±	0.03	0.11	±	0.03	0.1	±	0.1	0.11	±	0.03	0.03	±	0.01^µ^	0.04	±	0.01	0.3	±	0.1
	2019 (n = 10)	0.2	±	0.1	0.2	±	0.1	0.2	±	0.1	0.08	±	0.02	0.3	±	0.1	0.2	±	0.1	0.12	±	0.03	<LOQ			0.05	±	0.01	0.7	±	0.2
**KDF—Kołobrzeg-Darłowo fishery**	2014 (n = 10)	0.3	±	0.1	0.3	±	0.2	0.12	±	0.04	<LOQ			0.16	±	0.03	0.13	±	0.05	0.2	±	0.1	0.03	±	0.01	0.04	±	0.01	1.3	±	0.3
	2015 (n = 10)	0.2	±	0.2	<LOQ			<LOQ			<LOQ			0.3	±	0.1	0.2	±	0.1	0.2	±	0.1	0.04	±	0.01	0.1	±	0.03	1.1	±	0.2
	2016 (n = 10)	0.08	±	0.02	0.3	±	0.1	0.07	±	0.01	<LOQ			0.13	±	0.05	0.12	±	0.03	0.09	±	0.02	0.05	±	0.01	0.05	±	0.01	0.8	±	0.1
	2017 (n = 10)	0.8	±	0.7	0.34	±	0.03	<LOQ			0.12	±	0.02^µ^	0.27	±	0.05	0.15	±	0.03	0.09	±	0.03	<LOQ			0.2	±	0.1	0.9	±	0.2
	2018 (n = 10)	1.9	±	0.6	0.9	±	0.3	2	±	2	0.1	±	0.1	0.11	±	0.04	0.15	±	0.04	0.11	±	0.04	0.04	±	0.01	0.06	±	0.02	0.8	±	0.4
	2019 (n = 10)	0.4	±	0.3	0.5	±	0.3	0.4	±	0.3	0.1	±	0.1	0.4	±	0.2	0.2	±	0.1	0.10	±	0.04	0.11	±	0.02^µ^	0.08	±	0.03	0.6	±	0.3
**WF—Władysławowo fishery**	2014 (n = 10)	0.3	±	0.2	0.21	±	0.02	<LOQ			<LOQ			0.2	±	0.1	0.15	±	0.02	0.12	±	0.03	0.04	±	0.01	0.03	±	0.01^µ^	0.6	±	0.1
	2015 (n = 10)	0.2	±	0.1	<LOQ			<LOQ			<LOQ			0.2	±	0.1	0.3	±	0.1	0.2	±	0.1	<LOQ			0.07	±	0.01^µ^	1.0	±	0.3
	2016 (n = 10)	0.2	±	0.1	1.5	±	0.4	0.08	±	0.03	<LOQ			<LOQ			0.13	±	0.04	0.08	±	0.01	<LOQ			0.04	±	0.01	0.7	±	0.2
	2017 (n = 10)	0.3	±	0.3	0.2	±	0.1	0.09	±	0.02	0.08	±	0.01^µ^	0.2	±	0.1	0.2	±	0.1	0.09	±	0.02	0.05	±	0.01	0.2	±	0.1	0.9	±	0.2
	2018 (n = 10)	3	±	1	0.9	±	0.4	0.9	±	0.5	0.1	±	0.1	0.13	±	0.04	0.2	±	0.1	0.13	±	0.04	0.04	±	0.01	0.07	±	0.04	0.7	±	0.3
	2019 (n = 10)	0.7	±	0.8	0.8	±	0.6	0.7	±	0.4	0.2	±	0.1	0.7	±	0.3	0.6	±	1.0	0.2	±	0.1	0.04	±	0.01	0.08	±	0.04	0.9	±	0.4
**GG—Gulf of Gdansk**	2014 (n = 10)	0.2	±	0.1	0.12	±	0.03	<LOQ			0.4	±	0.3	0.5	±	0.4	0.4	±	0.4	0.2	±	0.1	0.04	±	0.01	0.03	±	0.02	0.7	±	0.2
	2015 (n = 10)	0.2	±	0.1	0.06	±	0.01	0.08	±	0.01^µ^	<LOQ			0.13	±	0.03	0.3	±	0.2	0.2	±	0.1	<LOQ			0.05	±	0.02	1.3	±	0.6
	2016 (n = 10)	0.14	±	0.04	<LOQ			0.08	±	0.02	<LOQ			0.2	±	0.2	0.4	±	0.5	0.4	±	0.3	<LOQ			0.05	±	0.02	0.9	±	0.4
	2017 (n = 10)	0.5	±	0.5	0.5	±	0.4	0.11	±	0.02^µ^	<LOQ			0.19	±	0.04	0.2	±	0.2	0.2	±	0.1	<LOQ			0.2	±	0.1	0.7	±	0.2
	2018 (n = 10)	0.3	±	0.3	0.3	±	0.1	0.2	±	0.1	<LOQ			0.11	±	0.05	0.10	±	0.05	0.11	±	0.05	0.03	±	0.01	0.07	±	0.04	1.0	±	0.4
	2019 (n = 10)	0.1	±	0.1	0.2	±	0.1	0.2	±	0.1	0.08	±	0.01	0.20	±	0.04	0.2	±	0.1	0.1	±	0.1	<LOQ			0.05	±	0.02	0.7	±	0.2
**VL— Vistula Lagoon**	2014 (n = 10)	0.4	±	0.1	0.2	±	0.2	0.3	±	0.2	0.2	±	0.2	0.2	±	0.2	0.4	±	0.3	0.2	±	0.2	0.2	±	0.2	0.2	±	0.2	2	±	1
	2015 (n = 10)	0.3	±	0.3	0.22	±	0.04^µ^	0.14	±	0.02^µ^	<LOQ			0.9	±	2.4	1.4	±	3.9	0.8	±	2.0	0.13	±	0.03	0.04	±	0.01	1.6	±	0.3
	2016 (n = 10)	0.10	±	0.03	0.15	±	0.03	<LOQ			<LOQ			<LOQ			0.22	±	0.05	0.3	±	0.1	<LOQ			<LOQ			2.9	±	0.6
	2017 (n = 10)	0.4	±	0.2	0.2	±	0.1	0.09	±	0.03	<LOQ			0.1	±	0.1	0.1	±	0.1	0.2	±	0.1	<LOQ			0.06	±	0.01	0.7	±	0.2
	2018 (n = 10)	0.4	±	0.2	0.2	±	0.1	0.2	±	0.1	0.09	±	0.02	0.19	±	0.04	0.10	±	0.03	0.19	±	0.04	0.1	±	0.1	0.03	±	0.01	0.5	±	0.2
	2019 (n = 10)	0.2	±	0.1	0.3	±	0.1	0.3	±	0.1	0.10	±	0.04	0.3	±	0.1	0.2	±	0.1	0.2	±	0.1	<LOQ			0.08	±	0.04	0.9	±	0.2

<LOQ—below the limit of quantification; µ—uncertainty of a single measurement.

**Table 3:** Min, Max and Range values of individual PFAS concentrations in perch.

Year (Number of Samples)			Concentration, µg/kg									
			PFBA	PFPeA	PFHxA	PFHpA	PFOA	PFNA	PFDA	PFBS	PFHxS	PFOS
**SL Szczecin Lagoon**	2014 (n = 10)	Min	0.2	0.1	0.1	0.3	0.2	0.3	0.4	0.04	0.04	1.9
		Max	0.6	0.5	0.1	0.3	0.7	4.2	2.0	0.1	0.04	6.4
		R	0.5	0.4	0.0	0.1	0.5	3.8	1.6	0.01	0.0	4.5
	2015 (n = 10)	Min	0.1	0.0	0.1	0.0	0.1	0.2	0.3	0.0	0.03	1.1
		Max	0.3	0.0	0.4	0.0	0.1	0.5	0.8	0.0	0.1	2.3
		R	0.3	0.0	0.2	0.0	0.0	0.3	0.5	0.0	0.0	1.2
	2016 (n = 10)	Min	0.1	0.1	0.1	0.0	0.1	0.3	0.6	0.0	0.03	3.9
		Max	0.1	0.2	0.1	0.0	0.1	0.5	1.5	0.0	0.05	7.2
		R	0.1	0.1	0.0	0.0	0.0	0.1	0.8	0.0	0.02	3.4
	2017 (n = 10)	Min	0.1	0.1	0.1	0.0	0.1	0.1	0.1	0.1	0.04	0.7
		Max	1.2	0.4	0.1	0.0	0.2	0.4	0.4	0.6	0.1	1.7
		R	1.1	0.3	0.1	0.0	0.2	0.3	0.3	0.5	0.1	1.0
	2018 (n = 10)	Min	0.1	0.1	0.1	0.1	0.3	0.1	0.3	0.03	0.03	1.2
		Max	0.3	0.2	0.2	0.1	0.9	0.2	0.9	0.03	0.03	2.7
		R	0.2	0.1	0.1	0.0	0.6	0.2	0.6	0.0	0.0	1.5
	2019 (n = 10)	Min	0.1	0.1	0.1	0.1	0.1	0.1	0.3	0.0	0.04	1.1
		Max	0.2	0.3	0.3	0.1	0.3	0.2	0.5	0.0	0.1	2.8
		R	0.2	0.2	0.2	0.0	0.2	0.1	0.2	0.0	0.1	1.7
**VLVistula Lagoon**	2014 (n = 10)	Min	0.2	0.1	0.1	0.1	0.1	0.1	0.1	0.0	0.0	0.6
		Max	0.5	0.7	0.7	0.5	0.8	0.9	0.7	0.5	0.4	4.5
		R	0.3	0.6	0.6	0.4	0.7	0.8	0.6	0.5	0.4	3.9
	2015 (n = 10)	Min	0.1	0.2	0.1	0.0	0.1	0.1	0.1	0.1	0.03	1.1
		Max	0.9	0.2	0.1	0.0	7.7	12.4	6.4	0.2	0.04	1.9
		R	0.8	0.0	0.0	0.0	7.6	12.3	6.3	0.0	0.01	0.8
	2016 (n = 10)	Min	0.1	0.1	0.0	0.0	0.0	0.1	0.2	0.0	0.0	2.3
		Max	0.2	0.2	0.0	0.0	0.0	0.3	0.4	0.0	0.0	4.3
		R	0.1	0.1	0.0	0.0	0.0	0.2	0.2	0.0	0.0	2.0
	2017 (n = 10)	Min	0.2	0.1	0.1	0.0	0.1	0.1	0.1	0.0	0.05	0.4
		Max	0.6	0.4	0.1	0.0	0.3	0.3	0.4	0.0	0.1	1.1
		R	0.5	0.3	0.1	0.0	0.2	0.2	0.3	0.0	0.03	0.7
	2018 (n = 10)	Min	0.3	0.1	0.1	0.1	0.1	0.1	0.1	0.03	0.03	0.2
		Max	1.0	0.3	0.4	0.1	0.2	0.1	0.2	0.2	0.04	1.0
		R	0.7	0.2	0.2	0.0	0.1	0.1	0.1	0.2	0.0	0.8
	2019 (n = 10)	Min	0.1	0.1	0.1	0.1	0.2	0.1	0.1	0.0	0.04	0.4
		Max	0.3	0.5	0.4	0.2	0.5	0.3	0.4	0.0	0.2	1.1
		R	0.2	0.4	0.3	0.1	0.3	0.2	0.2	0.0	0.1	0.8

**Table 4:** Min, Max and Range values of individual PFAS concentrations in herring.

Year (Number of Samples)			Concentration, µg/kg									
			PFBA	PFPeA	PFHxA	PFHpA	PFOA	PFNA	PFDA	PFBS	PFHxS	PFOS
**KDF Kołobrzeg** **—Darłowo fishery**	2014 (n = 10)	Min	0.2	0.2	0.1	0.0	0.1	0.1	0.1	0.03	0.03	0.7
		Max	0.4	0.6	0.2	0.0	0.2	0.2	0.3	0.04	0.1	1.8
		R	0.2	0.5	0.1	0.0	0.1	0.1	0.2	0.0	0.0	1.1
	2015 (n = 10)	Min	0.1	0.0	0.0	0.0	0.2	0.1	0.1	0.03	0.0	0.7
		Max	0.7	0.0	0.0	0.0	0.5	0.3	0.2	0.04	0.1	1.3
		R	0.6	0.0	0.0	0.0	0.3	0.2	0.2	0.01	0.1	0.6
	2016 (n = 10)	Min	0.1	0.2	0.1	0.0	0.1	0.1	0.1	0.04	0.03	0.6
		Max	0.1	0.4	0.1	0.0	0.2	0.2	0.1	0.1	0.1	1.0
		R	0.1	0.2	0.0	0.0	0.2	0.1	0.0	0.04	0.02	0.5
	2017 (n = 10)	Min	0.2	0.3	0.0	0.1	0.2	0.1	0.1	0.0	0.1	0.6
		Max	2.0	0.4	0.0	0.1	0.3	0.2	0.1	0.0	0.3	1.3
		R	1.8	0.0	0.0	0.0	0.1	0.1	0.1	0.0	0.2	0.7
	2018 (n = 10)	Min	1.2	0.3	0.3	0.1	0.1	0.1	0.1	0.03	0.04	0.4
		Max	2.9	1.2	7.0	0.2	0.2	0.2	0.2	0.1	0.1	1.6
		R	1.8	0.9	6.8	0.1	0.1	0.1	0.1	0.03	0.1	1.2
	2019 (n = 10)	Min	0.1	0.3	0.1	0.1	0.2	0.1	0.1	0.1	0.04	0.3
		Max	0.9	1.4	1.2	0.3	0.7	0.4	0.2	0.1	0.1	1.4
		R	0.8	1.1	1.1	0.2	0.5	0.3	0.1	0.0	0.1	1.1
**WF—Władysławowo fishery**	2014 (n = 10)	Min	0.2	0.2	0.0	0.0	0.1	0.1	0.1	0.03	0.03	0.5
		Max	0.7	0.2	0.0	0.0	0.3	0.2	0.2	0.04	0.03	0.7
		R	0.6	0.0	0.0	0.0	0.2	0.1	0.1	0.01	0.0	0.3
	2015 (n = 10)	Min	0.1	0.0	0.0	0.0	0.2	0.2	0.1	0.0	0.1	0.6
		Max	0.4	0.0	0.0	0.0	0.5	0.5	0.4	0.0	0.1	1.8
		R	0.3	0.0	0.0	0.0	0.3	0.3	0.3	0.0	0.0	1.2
	2016 (n = 10)	Min	0.1	1.0	0.1	0.0	0.0	0.1	0.1	0.0	0.0	0.5
		Max	0.5	2.4	0.1	0.0	0.0	0.2	0.1	0.0	0.1	0.9
		R	0.4	1.3	0.0	0.0	0.0	0.1	0.0	0.0	0.0	0.4
	2017 (n = 10)	Min	0.1	0.1	0.1	0.1	0.1	0.1	0.1	0.0	0.1	0.6
		Max	1.1	0.3	0.1	0.1	0.3	0.3	0.1	0.1	0.3	1.2
		R	1.0	0.2	0.0	0.0	0.2	0.2	0.0	0.0	0.2	0.6
	2018 (n = 10)	Min	0.7	0.4	0.2	0.1	0.1	0.1	0.1	0.03	0.03	0.4
		Max	5.4	1.7	1.8	0.3	0.2	0.4	0.2	0.1	0.1	1.3
		R	4.6	1.3	1.6	0.2	0.1	0.3	0.1	0.03	0.1	1.0
	2019 (n = 10)	Min	0.1	0.1	0.3	0.1	0.3	0.2	0.1	0.03	0.03	0.3
		Max	2.8	2.2	1.4	0.3	1.4	3.3	0.4	0.04	0.2	1.6
		R	2.7	2.1	1.1	0.3	1.1	3.1	0.4	0.0	0.1	1.3

**Table 5:** Min, max and range values of individual PFAS concentrations in flatfish.

Year (Number of Samples)			Concentration, µg/kg									
			PFBA	PFPeA	PFHxA	PFHpA	PFOA	PFNA	PFDA	PFBS	PFHxS	PFOS
**PB—** **Pomeranian Bay**	2014 (n = 10)	Min	0.1	0.1	0.1	0.0	0.1	0.1	0.2	0.0	0.03	0.3
		Max	0.2	0.4	0.2	0.0	0.2	0.3	0.4	0.0	0.1	0.5
		R	0.1	0.3	0.1	0.0	0.1	0.2	0.2	0.0	0.04	0.2
	2015 (n = 10)	Min	0.1	0.1	0.1	0.0	0.1	0.1	0.2	0.03	0.03	0.5
		Max	0.3	0.1	0.2	0.0	0.5	1.2	1.2	0.03	0.04	0.7
		R	0.2	0.0	0.1	0.0	0.4	1.1	1.0	0.0	0.0	0.3
	2016 (n = 10)	Min	0.1	0.1	0.1	0.0	0.0	0.1	0.1	0.0	0.0	0.5
		Max	0.2	0.6	0.1	0.0	0.0	0.3	0.4	0.0	0.0	1.3
		R	0.1	0.5	0.0	0.0	0.0	0.2	0.2	0.0	0.0	0.8
	2017 (n = 10)	Min	0.2	0.2	0.2	0.0	0.1	0.1	0.1	0.0	0.04	0.2
		Max	2.0	0.3	0.3	0.0	0.2	0.2	0.7	0.0	0.1	0.5
		R	1.8	0.2	0.2	0.0	0.2	0.2	0.6	0.0	0.1	0.3
	2018 (n = 10)	Min	0.2	0.1	0.2	0.1	0.1	0.1	0.1	0.03	0.04	0.2
		Max	0.4	0.4	0.3	0.1	0.2	0.2	0.2	0.03	0.05	0.5
		R	0.2	0.2	0.1	0.1	0.1	0.1	0.1	0.0	0.0	0.3
	2019 (n = 10)	Min	0.1	0.1	0.1	0.1	0.1	0.1	0.1	0.0	0.04	0.4
		Max	0.3	0.3	0.3	0.1	0.5	0.5	0.2	0.0	0.1	1.3
		R	0.2	0.2	0.2	0.1	0.4	0.4	0.1	0.0	0.04	0.9
**GG -Gulf of Gdansk**	2014 (n = 10)	Min	0.1	0.1	0.0	0.1	0.1	0.1	0.1	0.03	0.03	0.4
		Max	0.4	0.2	0.0	0.7	1.5	1.1	0.4	0.1	0.03	0.9
		R	0.2	0.1	0.0	0.6	1.3	0.9	0.2	0.02	0.0	0.5
	2015 (n = 10)	Min	0.1	0.1	0.1	0.0	0.1	0.1	0.1	0.0	0.0	0.7
		Max	0.3	0.1	0.1	0.0	0.2	0.7	0.3	0.0	0.1	2.5
		R	0.2	0.0	0.0	0.0	0.1	0.6	0.2	0.0	0.0	1.8
	2016 (n = 10)	Min	0.1	0.0	0.1	0.0	0.1	0.1	0.1	0.0	0.03	0.5
		Max	0.2	0.0	0.1	0.0	0.5	1.7	1.2	0.0	0.1	1.8
		R	0.1	0.0	0.0	0.0	0.5	1.6	1.1	0.0	0.1	1.3
	2017 (n = 10)	Min	0.1	0.1	0.1	0.0	0.2	0.1	0.1	0.0	0.1	0.4
		Max	1.5	1.2	0.1	0.0	0.3	0.7	0.3	0.0	0.5	1.1
		R	1.4	1.1	0.0	0.0	0.1	0.6	0.2	0.0	0.4	0.7
	2018 (n = 10)	Min	0.2	0.1	0.1	0.0	0.1	0.1	0.1	0.03	0.03	0.5
		Max	1.1	0.5	0.3	0.0	0.2	0.2	0.2	0.03	0.1	1.7
		R	0.9	0.4	0.2	0.0	0.2	0.1	0.2	0.0	0.1	1.2
	2019 (n = 10)	Min	0.1	0.1	0.1	0.1	0.2	0.1	0.1	0.0	0.03	0.5
		Max	0.3	0.3	0.6	0.1	0.3	0.3	0.3	0.0	0.1	1.2
		R	0.3	0.2	0.5	0.0	0.1	0.2	0.2	0.0	0.0	0.6

**Table 6:** Summary of native and mass-labelled standards [62].

Compound Name	Acronym	CAS Number	Monoisotopic Mass, g/mol
Perfluorobutanoic acid	PFBA	375-22-4	213.986
Perfluoropentanoic acid	PFPA	2706-90-3	263.983
Perfluorohexanoic acid	PFHxA	307-24-4	313.980
Perfluoroheptanoic acid	PFHpA	375-85-9	363.977
Perfluorooctanoic acid	PFOA	335-67-1	413.974
Perfluorononanoic acid	PFNA	375-95-1	463.970
Perfluorodecanoic acid	PFDA	335-76-2	513.967
Perfluorobutanesulfonic acid	PFBS	375-73-5	299.950
Perfluorohexanesulfonic acid	PFHxS	355-46-4	399.944
Perfluorooctanesulfonic acid	PFOS	1763-23-1	499.937
Perfluoro-n-[1,2,3,4-13C4]butanoic acid	MPFBA	1017281-29-6	217.999
Perfluoro-n-[1,2-13C2]hexanoic acid	MPFHxA	960315-47-3	815.954
Perfluoro-n-[1,2,3,4-13C4]octanoic acid	MPFOA	960315-48-4	417.987
Perfluoro-n-[1,2,3,4,5-13C5]nonanoic acid	MPFNA	960315-49-5	468.987
Perfluoro-n-[1,2-13C2]decanoic acid	MPFDA	960315-52-0	615.968

## Data Availability

Data are contained within the article.
